# Galectin-3 is associated with the functional outcome and mortality in stroke patients: A systematic review and meta-analysis

**DOI:** 10.1016/j.heliyon.2023.e13279

**Published:** 2023-01-26

**Authors:** Xiaoling Han, Bingbing Geng, Feiyan Deng, Ying Ma, Ningning Fan, Shaomin Huang, Ming Xue, Lei Wu, Bixia Li, Shaoqin Liao, Qiao Ye, Yu Liu

**Affiliations:** a Interventional Medical Center, Zhuhai People’s Hospital (Zhuhai People’s Hospital of Jinan University), Zhuhai, Guangdong, China; b Ophthalmology Department, Zhuhai People’s Hospital, Zhuhai, Guangdong, China; c Nursing Department, Zhuhai People’s Hospital, Zhuhai, Guangdong, China

**Keywords:** Stroke, Galectin-3, Prognosis, Meta-analysis, Functional outcome

## Abstract

**Introduction:**

There is still a lack of sensitive predictive tools for stroke outcomes. High galectin-3 concentration is associated with an increased risk of stroke. This study investigated the relationship between blood galectin-3 levels and stroke prognosis.

**Methods:**

The PubMed, EMBASE, and Cochrane Library databases were searched as of May 2021. Data from eligible studies on the relationship between galectin-3 and stroke prognosis were extracted for the meta-analysis.

**Results:**

The outcomes included the modified Rankin Scale (mRS), mortality rate, and prognostic accuracy of galectin-3 on mRS after stroke. Odds ratio (OR) and 95% CI were used to assess the association between galectin-3 and the prognostic outcomes. Subgroup analysis based on the study design was performed to evaluate the correlation of galectin-3 with mRS and mortality. A random-effects model was adopted for this meta-analysis. A total of 5 studies involving 3607 stroke patients were included. Higher serum galectin-3 level was associated with mRS (OR [95% CI]: 2.02 [1.08, 3.77]) and mortality (OR [95% CI]: 2.17 [1.17, 4.02]) after stroke. Subgroup analysis revealed a similar relationship between galectin-3 and mRS for both prospective and retrospective studies. There were no associations between galectin-3 level and mortality rate in prospective studies. Galectin-3 had a good predictive ability on mRS after stroke (AUC: 0.88, 95% CI:0.85, 0.91).

**Conclusion:**

Elevated blood galectin-3 levels were associated with prognostic outcomes after stroke, including functional outcome mRS and mortality rate. Moreover, galectin-3 had a good predictive ability for the prognosis of stroke.

## 1 Introduction

Stroke patients will increase by 27% between 2017 and 2047 due to an aging population and improved survival rates [1]. Although ischemic stroke is the major type of these cases [2,3], the various etiology and clinical progress of cerebral ischemia make it difficult to predict stroke prognostication [[4], [5], [6]], highlighting the need for more sensitive predictive tools and biomarkers [[7], [8], [9]]. Previous studies have emphasized the potential of blood biomarkers in predicting functional outcomes and mortality after stroke [[10], [11], [12], [13]].

Galectin-3 (Gal-3) is a member of the galectin family with a binding affinity for β-galactosides [14]. Beta-galactoside is located within the cytoplasm and the nucleus but can be transported to the cell surface and extracellular space and into blood circulation. Gal-3 plays important roles in the cellular process, including cell proliferation, adhesion, differentiation, angiogenesis, and apoptosis [15]. Besides, mounting evidence shows that Gal-3 may act as a mediator of tumor cell transformation, migration, invasion, and metastasis [16].

Gal-3 has been implicated in the pathogenesis of ischemic brain injuries. Research has increasingly suggested that Gal-3 level is significantly elevated in microglia during neonatal hypoxic-ischemic brain injury, contributing to hypoxic-ischemic brain injury [17]. Additionally, Gal-3 mediates post-ischemic tissue remodeling. Exogenous Gal-3 increases endothelial and neural progenitor cell proliferation, and enhances microvessel density in ischemic rat brains [18]. Although many studies have investigated the association between Gal-3 and ischemic stroke prognosis in the clinical setting, the conclusion remains unclear [[19], [20], [21], [22], [23]]. Zhuang et al. showed that higher serum levels of Gal-3 were related to stroke severity at admission and stroke prognosis at discharge [24]. Hansen et al. suggested that the correlation of Gal-3 with stroke mortality changed over time [25]. The study by Zeng et al. found that increased serum Gal-3 level was linked with stroke recurrence and vascular events within 1 year after stroke in patients with hyperglycemia [23].

Moreover, Gal-3 has recently attracted significant attention for its prognostic value in stroke [20,25,26]. Many of the studies were small-scale research with inherent weaknesses in qualities. The clinical usefulness of blood Gal-3 in predicting the prognosis of ischemic stroke has yet to be established. The present study was the first comprehensive review to explore the relationship between stroke prognosis and Gal-3, and determine whether gal-3 could be served as a potential prognostic biomarker for stroke.

## 2 Materials and methods

This systematic review and meta-analysis was performed according to the Preferred Reporting Items for Systematic Reviews and Meta-Analyses (PRISMA) guideline [27].

### 2.1 Eligibility criteria

This review included articles relevant to the association between Galectin-3 and the functional outcome and mortality in stroke patients and met the following eligibility criteria:

(1) Population: patients with first-ever ischemic stroke confirmed by computed tomography or magnetic resonance imaging were enrolled.
(2) Outcome: all patients were offered the blood sample test of serum Gal-3 level. The functional outcome of included patients was assessed by the mRS, and the safety outcome was measured by the mortality rate.
(3) Study design: eligible RCTs and observational studies were included regardless of study design.
(4) Language: the language of the included studies was restricted to English.

The studies that met the following criteria for exclusion from this review would not be included:

(1) Insufficient information on study design, population, outcome, or exposure.
(2) Sample size <10.
(3) Duplicate results.
(4) Case reports, case series, in vitro studies, literature reviews, and other studies against the inclusion criteria.

### 2.2 Search strategy

Two independent examiners (Xiaoling Han and Bingbing Geng) performed a literature search in PubMed, Embase, and Cochrane Library. We kept the publication date open to avoid omitting unpublished or ongoing research. The search strategy included the keywords transformation, and we modified the search strategy based on the rules of each database. MeSH terms of ‘Galectin 3’, ‘Stroke’, relevant keywords, as well as Boolean operators (OR, AND) were used to locate the eligible studies. The language was limited to English, and articles were searched up to May 2021. Duplicates were deleted based on EndNote X9. We resolved all discrepancies through discussion.

EndNote X9 was used to organize the search outcomes and conduct the titles and abstract screening. After removing the duplicates, two independent reviewers (Feiyan Deng and Ying Ma) screened the titles and abstracts for articles that met the inclusion criteria. The full-text screening included the potentially eligible studies and articles without available abstracts or abstracts that did not have sufficient judgment evidence. All discrepancies in the screening results were resolved through discussion until a consensus was reached.

### 2.3 Data extraction

The extracted data comprised authors, year of publication, the country where the study was performed, study design, patient characteristics (i.e., sex, NIHSS score, mRS, Gal-3 at admission, and follow-up duration). The outcome data included functional outcomes represented with mRS and mortality rate after stroke.

### 2.4 Quality of the evidence

A total of five studies entered our final meta-analysis model. The level of evidence of all articles was assessed independently by two authors according to the Newcastle-Ottawa Scale (NOS) criteria for quality assessment of Cohort studies and case control, Version 2 of the Cochrane risk-of-bias assessment tool for randomized trials. Differences of opinion among authors are discussed to reach consensus.

### 2.5 Statistical analysis

All the analyses were performed using the STATA SE 14.0 software (StataCorp, College Station, Texas, USA). Statistical heterogeneity among these studies was calculated by Cochran’s Q test and the I^2^ index (over 50%, and P < 0.1, high heterogeneity). A random-effects model was applied based on the heterogeneity test. OR with 95% CI was used to evaluate the association between Gal-3 and prognostic outcomes. A P value less than 0.05 was considered statistically different. Potential publication bias was assessed by funnel plots and Egger’s test, as appropriate given the known limitations that the funnel plots and Egger’s test could yield misleading results in meta-analysis with small numbers of studies [26].

## 3 Results

### 3.1 Study selection and characteristics of included studies

**Fig. 1:**
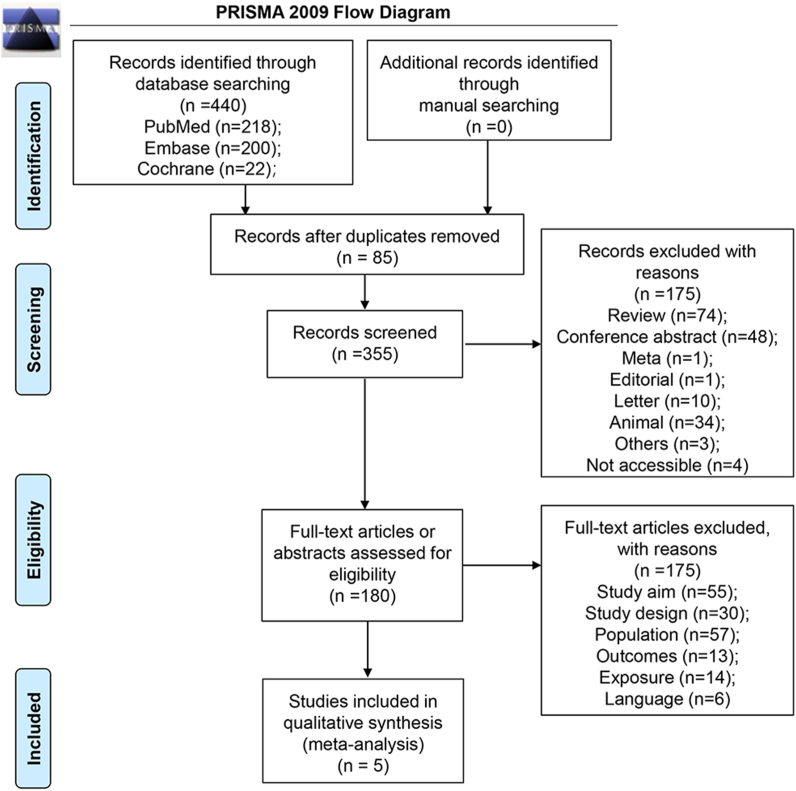
Flow diagram of the study selection process.

Five studies with 3607 ischemic stroke patients were included in the meta-analysis. Four hundred forty relevant publications were initially identified during the database search. Duplicated documents, reviews, conference abstracts, meta-analyses, editorials, letters, animal studies, inaccessible publications, and other ineligible studies were removed. After the full-text screening, 175 documents were excluded due to insufficient information on study design, population, outcome, exposure, and language limitation. The PRISMA flowchart is illustrated in Fig. 1.

The characteristics of the five studies, including one RCT and four observational studies, are listed in Table 1. A total of 3607 patients were enrolled. Three studies came from China, one study from the USA, and one study contained mixed populations from China and US. The patients had a mean age of 62.4–71.2 years old. The follow-up time was 3 months to 1 year. Three studies record the modified Rankin Scale (mRS). Gal-3 concentration at admission was 7.3–7.9 ng/ml. However, two studies did not mention the specific number of Gal-3 level; they were still included because the association between Gal-3 and ischemic stroke patients' mortality rate were identified.

**Table 1:** Literature search and characteristics of the included studies.

Study	Design	Country	Sample size	Age (years), mean (SD)/median (IQR)	Male sex, n (%)	NIHSS score	mRS	Gal - 3 (ng/ml) at admission	Follow up duration of mRS
Liu 2020	RCT	China, USA	2694	62.4 (10.8)	1714 (63.62)	4 (3, 7)	/	/	1 year
Yan 2016	Prospective observational study	China	112	66 (58.9)	63.2 (9.6)	11 (7)	/	/	6 months
Zhuang 2021	Prospective observational study	China	288	67 (59–74)	167 (58)	6 (4–12)	1 (0–3)	7.3 (5.1–11.6)	/
Hansen 2020	Retrospective cohort study	USA	383	71 (59, 81)	227 (57%)	6 (3,12)	2 (1,4)	7.4 (5.2–11.7)	90 days
He 2017	Case - control	China	130	71.2 (9)	79 (60.8)	4 (2,8)	3 (2,4)	7.9 (7,9)	90 days

*Abbreviation:* NIHSS, National Institutes of Health Stroke Scale; mRS, modified Rankin Scale.

### 3.2 Quality and risk of bias assessment results

The overall methodological quality scores for three cohort studies ranged from 5 to 7 based on NOS criteria, indicating low to moderate bias (Supplementary Table 1a). According to ROB 2.0 quality assessment, only the RCT by Liu et al. had a low bias about intervention and outcome assessment, but the selection and reporting bias were unclear (Supplementary Table 1b). The case-control study by He et al. was rated high, with an overall quality score of 7 (Supplementary Table 1c). Publication bias was not assessed as there were inadequate numbers of included trials to assess a funnel plot or more advanced regression-based assessments properly.

### 3.3 Association between Gal-3 and mRS after stroke

**Fig. 2:**
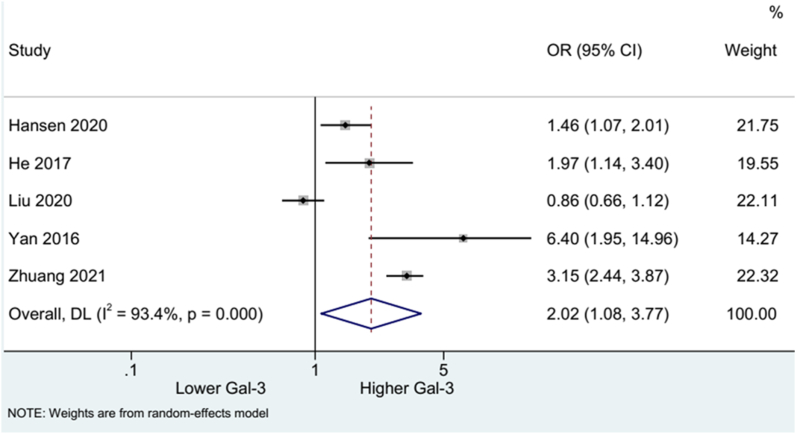
Forest plot of mRS.

**Fig. 3:**
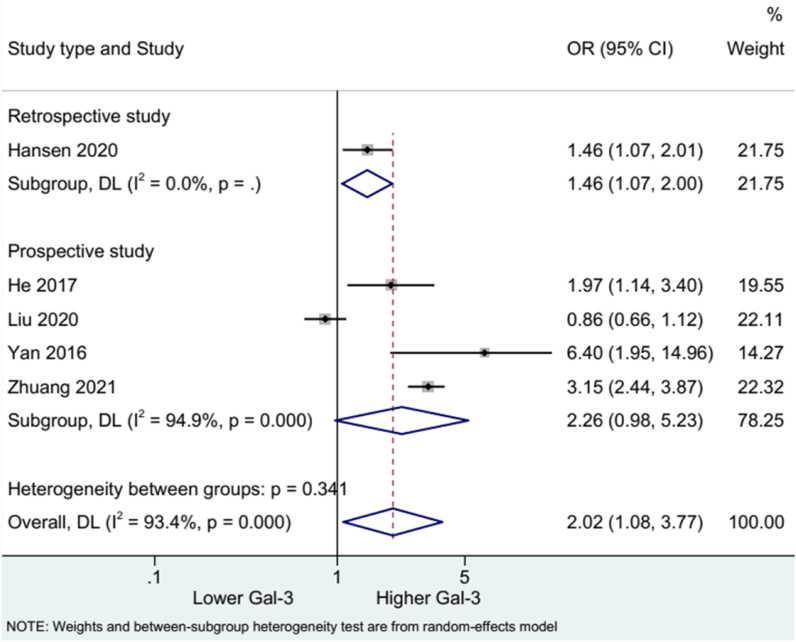
Forest plot of mRS by study type.

Five studies were included to evaluate mRS after stroke. The pooled results using the random-effects model showed higher blood Gal-3 concentration was associated with increased risk of mRS (OR: 2.02, 95%CI:1.08, 3.77). High heterogeneity was detected across the studies (I^2^ = 93.4%, p = 0.000) (Fig. 2). Subgroup analysis stratified by study design suggested the correlation between high Gal-3 level and mRS risk in four prospective (OR: 2.26, 95%CI:0.98, 5.23) and one retrospective study (OR: 1.46, 95%CI:1.07, 2.00). High heterogeneity was observed among the prospective studies (I^2^ = 94.9%, p = 0.000) (Fig. 3).

### 3.4 Association between Gal-3 and mortality

The data on the mortality outcome of stroke patients from three studies were incorporated for the pooled analysis. The results showed that a high Gal-3 level was linked with a higher mortality rate in stroke (OR: 2.17, 95% CI: 1.17, 4.02). Substantial heterogeneity was found (I^2^ = 66.2%, p = 0.052) (Fig. 5). Subgroup analysis of one retrospective study showed a similar relationship between high Gal-3 levels and mortality (OR: 2.17, 95% CI: 1.17, 4.02). Two prospective studies revealed no association between Gal-3 level and mortality rate (OR: 2.17, 95% CI: 1.17, 4.02; I^2^ = 82.6%, p = 0.016) (Fig. 6).

### 3.5 Predictive value of Gal-3 on mRS after stroke

**Fig. 4:**
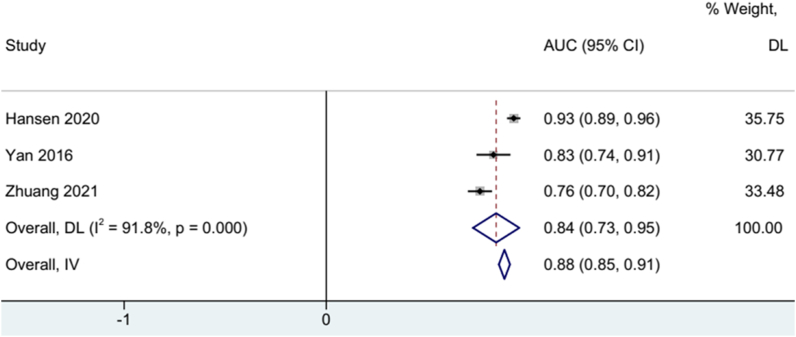
Forest plot of AUC to evaluate the accuracy of galectin-3 to diagnose mRS after stroke.

Three studies use the Receiver Operating Characteristic (ROC) curve to assess the predictive value of Gal-3 on mRS after stroke. Therefore we extracted Area Under Curve (AUC) value to represent the capability of Gal-3 to predict mRS after stroke. The pooled result revealed a good predictive ability of Gal-3 on mRS (AUC: 0.88, 95% CI:0.85, 0.91). A random-effects model was used based on high heterogeneity test (I^2=^91.8%, p = 0.000) (Fig. 4).

**Fig. 5:**
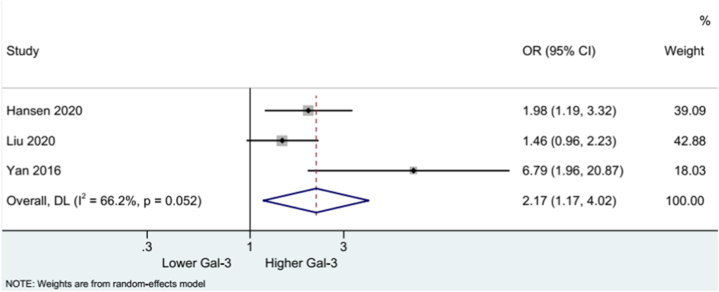
Forest plot of mortality.

**Fig. 6:**
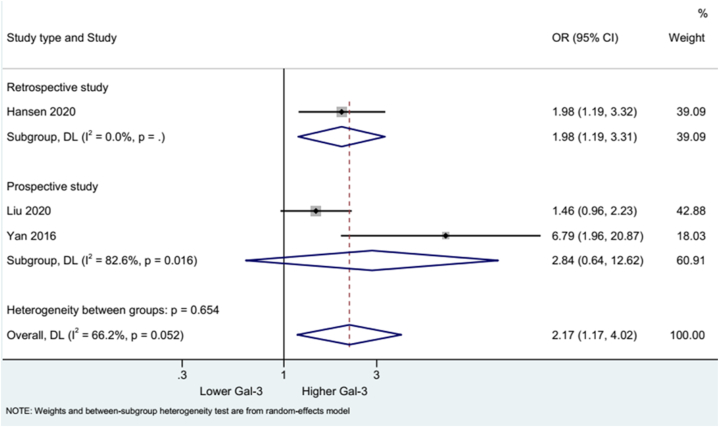
Forest plot of mortality by study type.

### 3.6 Sensitivity analysis

**Fig. 7:**
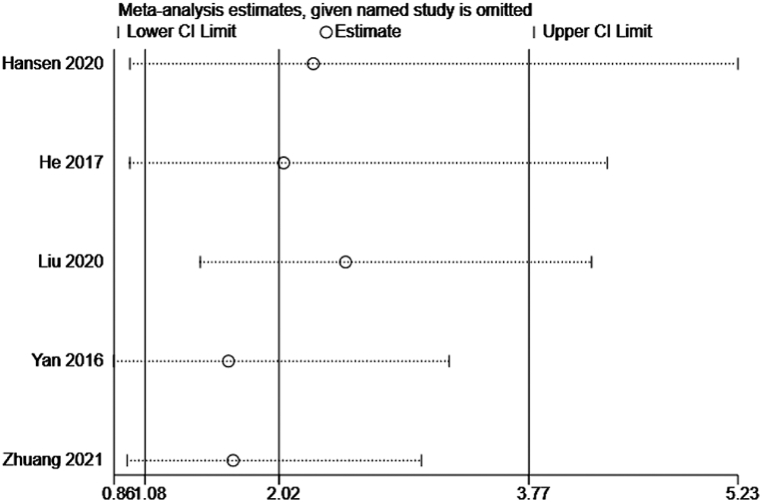
Sensitivity analysis of mRS.

**Fig. 8:**
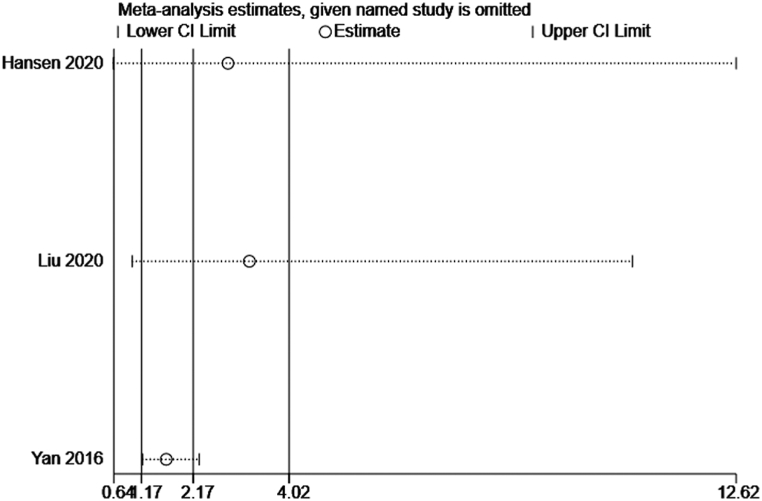
Sensitivity analysis of mortality.

Sensitivity analysis is a tool to determine the robustness of an assessment by examining the impact of key variations, such as methods, variables, outcomes, and assumptions, on the overall conclusions. We used leave-one-out method in the sensitivity analysis on the results of mRS and mortality. The findings demonstrated that the pooled results on mRS and mortality were reliable (Figs. 7 and 8). However, when analyzing the association between Gal-3 and mRS, the Liu (2020) results may lead to the possible source of heterogeneity because this study identified the combined effect of NT-proBNP and galectin-3 on ischemic stroke patients' clinical outcomes rather than Gal-3 alone.

## 4 Discussion

Developing useful blood biomarkers for predicting stroke prognosis is of great significance for the early prevention and treatment of this disease. To the best of our knowledge, this study was the first systemic review and meta-analysis to propose Gal-3 as a potential biomarker for predicting the clinical prognosis of stroke patients. The primary outcome of our meta-analysis showed that higher serum levels of Gal-3 were associated with increased mRS and mortality rate after stroke. The exploratory outcome identified that elevated Gal-3 levels had a good predictive value for the poor prognosis of stroke patients.

Gal-3 functions in cancer and cardiovascular diseases through the modulation of angiogenic and apoptotic pathways, inflammation, oxidative stress, and cardiac remodeling. The neuro-vascular protective effects of Gal-3 have also been proposed, which may be involved in functional recovery after ischemic stroke. Studies have shown that GAL-3 is involved in the pathological process of atherosclerosis [28], such as its upregulation in human atherosclerotic plaques [29], and its beneficial effects after inactivation of the GAL-3 gene [30] have been reported in animal studies. In the apolipoprotein E− (ApoE-) -deficient mouse model, the number of atherosclerotic lesions and perivascular inflammatory infiltration [30] were reduced in mice with GAL-3 gene knockout compared with mice with active GAL-3 expression.

Based on these possible mechanisms of Gal-3 as a biomarker for prognosis in stroke patients and the evidence from animal trial studies, this review focused on the influence of Gal-3 on stroke functional outcomes indicated by mRS and mortality outcome. In the study by He et al., higher Gal-3 level was found to be independently associated with an unfavorable outcome (mRS > 2) on day 90 after ischaemic stroke during large artery atherosclerotic (LAA) stroke, after adjusting for clinical variables including age, sex, body mass index, hypertension, diabetes, dyslipidemia, smoking, drinking, homocysteine, creatinine, and hs-CPR [31]. Dong et al. found that increased serum Gal-3 levels were associated with poor mRS functional score at admission and poor outcome after a 1-year follow-up, yet similar Gal-3 level was seen between patients with good (mRS score: 0–2 points) and bad recoveries (mRS score: 3–6 points) [20]. Contradictory results were also reported. Liu et al. assessed the major disability through mRS and found that the distribution of mRS in the high NT-proBNP/low galectin-3 stroke group was worse than that in the low NT-proBNP/high galectin-3 stroke group [32]. Our pooled analysis results suggested that elevated serum Gal-3 concentration might be associated with poor functional outcomes in stroke patients. However, this result is only based on a small number of studies. Further results from RCTs with larger sample sizes addressing this issue are needed to ensure more adequate and reliable evidence.

Hansen et al. showed that Gal-3 concentration measured at the time of admission was associated with long-term poor functional outcomes and 90-day mortality (OR 1.98, 95% CI 1.19–3.32, P = 0.009) in acute stroke patients. Liu et al. also observed a marginally significant association of high galectin-3 levels with a 1-year mortality rate [32]. Our meta-analysis consistently showed that increased serum Gal-3 level was linked with a lower mortality rate in stroke patients. Hansen et al. identified the harmful effects of elevated GAL-3 in the early acute phase of stroke [25]. Other study results suggested that GAL3-dependent TLR4 activation can promote the sustained activation of microglia and prolong the inflammatory response in the brain. These are all the potential reasons for the influence of GAL-3 on mortality.

As an exploratory outcome, this meta-analysis tries to summarize the accuracy of Gal-3 as a biomarker to predict mRS after stroke. And the pooled result revealed its good predictive ability. Previous studies have already proposed some factors affecting the prediction effect of GAL-3 on mRS and several feasible prediction models. Dong et al. performed ROC analysis to assess the predictive ability of Gal-3 to distinguish patients with good recovery from those with bad recovery [20]. They found that the cut-off value of 53.5 ng/ml Gal-3 exhibited good predictive power on functional outcomes of AIS patients with an AUC of 0.884, a sensitivity of 88.4%, and a specificity of 76.9%. The timing of blood sample collection affected the predictive ability of Gal-3 in stroke. Hansen et al. showed that increased Gal-3 level at admission was able to predict 90-day functional outcomes (AUC: 0.673) and mortality (AUC: 0.724) in acute stroke patients, and the predictions performed better when measured at admission than at 48 h after stroke, indicating time-dependence of Gal-3 prediction [25].

Additionally, gal-3 levels combined with NIHSS scores or hematoma volumes significantly improved AUCs for predicting 6-month mortality and 6-month unfavorable outcome. According to the ROC analysis conducted by Zhuang et al., Gal-3, under the optimal threshold of 8.6 ng/ml, showed the best predictive effect for the adverse functional outcome defined by the Youden Index compared with CRP and age but similar to the predictive value of the NIHSS score [24]. Several studies have shown that Gal-3 combined with NT-proBNP or HDL-C resulted in a more accurate predicted prognosis of ischemic stroke [32]. Moreover, hyperglycemia may modify the prognostic value of Gal-3 after ischemic stroke [23].

Significant heterogeneities were detected for the outcomes of mRS, mortality, and prognostic ability of Gal-3 in stroke. Study design, age, gender, BMI, baseline NIHSS scores, follow-up time, history of diseases, and medications might be associated with the heterogeneity. In several included studies, multivariate regression analyses were performed to reduce the heterogeneity.

Several limitations should be noted for the present study. First, this meta-analysis mainly comprised observational studies, which had limitations in selection and outcome assessment, and caution should be taken in interpreting the results. Second, different cut-off values of gal-3 were used when using ROC analysis to evaluate its predictive power. Third, the timing of blood Gal-3 sampling varied among the studies. Furthermore, the generalization of the findings from this study should be extrapolated carefully to other populations because the majority of included studies were carried out in China. Also, with such a small number of analyzed trials, all the methods for possible detection of publication bias are underpowered.

This meta-analysis showed that higher circulating Gal-3 levels are associated with functional outcomes and survival of stroke patients. Gal-3 could be a useful prognostic biomarker to predict the prognosis of stroke. A further large sample and rigorously designed studies were warranted to generalize our findings. Future studies to lower the galectin-3 level may provide a new direction to reduce disease severity and improve the prognosis of stroke.

## Declarations

### Author contribution statement

All authors listed have significantly contributed to the development and the writing of this article.

### Funding statement

Dr Yu Liu was supported by 10.13039/501100003453Natural Science Foundation of Guangdong Province [2019A1515010279].

Shaoqin Liao was supported by Zhuhai Science and Technology Planning Project [ZH22036201210179PWC].

### Data availability statement

Data included in article/supp. material/referenced in article.

### Declaration of interest’s statement

The authors declare no competing interests.
