# Supplementary material for: Galectin-3 is associated with the functional outcome and mortality in stroke patients: A systematic review and meta-analysis
Source: Heliyon. 2023 Jan 26;9(2):e13279. doi: 10.1016/j.heliyon.2023.e13279 (PMC9947260; doi:10.1016/j.heliyon.2023.e13279)
Supplement: Multimedia component 1 [file mmc1.docx]

Supplementary information

Search strategy (Query in PubMed as an example):

("Galectin 3"[Title/Abstract] OR "Galectin-3"[Title/Abstract] OR Gal-3[Title/Abstract] OR Galectin 3[MeSH]) AND (stroke[Title/Abstract] OR stroke[MeSH])
